# Supplementary material for: COVID-19: exploring impacts of the pandemic and lockdown on mental health of Pakistani students
Source: PeerJ. 2021 Feb 3;9:e10612. doi: 10.7717/peerj.10612 (PMC7866897; doi:10.7717/peerj.10612)
Supplement: Supplemental Information 2 [file peerj-09-10612-s002.docx]

**IMPACT OF COVID-19 AND LOCK-DOWN ON UNIVERSITY STUDENTS IN PAKISTAN**

Dear students,
You are invited to participate in this cross-sectional survey, which is designed to assess anxiety, coping strategy and behavioural pattern among university students in Pakistan during this COVOD-19 and Lock-down period.

Participation in this study is voluntary. We assure that information provided you will be PRIVATE AND CONFIDENTIAL. Your information will only be used for the purpose of this research

With this, our team thanks you for your participation in this study. Any doubts or inquiries you can email the primary investigator at [drgulbuledi@gmail.com](about:blank). Have a nice day

**Before we start**

Consent to participate *

I have read the statement above, understood the same, and voluntarily agree to participate in the study.

**Please tell us about yourself**

Gender *

Male

Female

Age *

Below 18 years

19-25 years

26-32 years

33-40 years

Above 40 years

Name of University *

Your answer

Field of Study (e.g. Management, Science, etc) *

Your answer

Level of Study *

Diploma

Undergraduate

Masters

PhD

Professional

Other:

Year of Study *

Year 1

Year 2

Year 3

Year 4

Year 5

Above 5 years

Nationality (e.g. Pakistani, Saudi, etc) *

Your answer

Ethnicity *

Sindhi

Balouchi

Pathans

Punjabi

Arab

African

Other:

Has your institution moved to virtual instruction due to COVID-19? *

Yes

No

Not Applicable

If Yes, When? *

January

February

March

April

Not Applicable

Has your institution closed dormitories/residencies/hostels due to COVID-19? *

Yes

No

Not Applicable

Where are you currently staying? *

College residency/hostel

Rented house outside campus

Rented apartment outside campus

Family Home

Relative's Home

Other:

**Please tell us how you feel during this COVID-19 pandemic**

If you are on a mobile phone, please rotate your display to see the full table

*Never or very rare Sometimes Often Very often or always

I feel more nervous and anxious than usual

I feel afraid for no reason at all

I get upset easily or feel panicky

I feel like I’m falling apart and going to pieces

I feel nothing is all right and something bad will happen

My arms and legs shake and tremble

I am bothered by headaches, neck and back pain

I feel weak and get tired easily

I do not feel calm and cannot sit still easily

I can feel my heart beating fast

I am bothered by dizzy spells

I have fainting spells or feel like it

I cannot breathe in and out easily

I get feelings of numbness and tingling in my fingers and toes

I am bothered by stomach-aches or indigestion

I go to washroom to pass urine often

My hands are usually wet and cold

My face gets hot and flushes

I cannot fall asleep easily and get a good nights’ rest

I have nightmares

I feel more nervous and anxious than usual

I feel afraid for no reason at all

I get upset easily or feel panicky

I feel like I’m falling apart and going to pieces

I feel nothing is all right and something bad will happen

My arms and legs shake and tremble

I am bothered by headaches, neck and back pain

I feel weak and get tired easily

I do not feel calm and cannot sit still easily

I can feel my heart beating fast

I am bothered by dizzy spells

I have fainting spells or feel like it

I cannot breathe in and out easily

I get feelings of numbness and tingling in my fingers and toes

I am bothered by stomach-aches or indigestion

I go to washroom to pass urine often

My hands are usually wet and cold

My face gets hot and flushes

I cannot fall asleep easily and get a good nights’ rest

I have nightmares

**Please tell us how you feel during this COVID-19 pandemic**

If you are on a mobile phone, please rotate your display to see the full table

*Never or very rare Sometimes Often Very often or always

I feel more nervous and anxious than usual

I feel afraid for no reason at all

I get upset easily or feel panicky

I feel like I’m falling apart and going to pieces

I feel nothing is all right and something bad will happen

My arms and legs shake and tremble

I am bothered by headaches, neck and back pain

I feel weak and get tired easily

I do not feel calm and cannot sit still easily

I can feel my heart beating fast

I am bothered by dizzy spells

I have fainting spells or feel like it

I cannot breathe in and out easily

I get feelings of numbness and tingling in my fingers and toes

I am bothered by stomach-aches or indigestion

I go to washroom to pass urine often

My hands are usually wet and cold

My face gets hot and flushes

I cannot fall asleep easily and get a good nights’ rest

I have nightmares

I feel more nervous and anxious than usual

I feel afraid for no reason at all

I get upset easily or feel panicky

I feel like I’m falling apart and going to pieces

I feel nothing is all right and something bad will happen

My arms and legs shake and tremble

I am bothered by headaches, neck and back pain

I feel weak and get tired easily

I do not feel calm and cannot sit still easily

I can feel my heart beating fast

I am bothered by dizzy spells

I have fainting spells or feel like it

I cannot breathe in and out easily

I get feelings of numbness and tingling in my fingers and toes

I am bothered by stomach-aches or indigestion

I go to washroom to pass urine often

My hands are usually wet and cold

My face gets hot and flushes

I cannot fall asleep easily and get a good nights’ rest

I have nightmares

**During this COVID-19 and Lock-down period, do you…**

If you are on a mobile phone, please rotate your display to see the full table

*Not applicable Less than before Same as before More than before

Eat fruits

Eat vegetables

Eat rice

Eat bread

Eat meat

Eat sea food

Eat noodles

Eat junk food

Drink water

Drink coffee/tea

Drink carbonated drinks

Smoke

Exercise

Sleep

Eat fruits

Eat vegetables

Eat rice

Eat bread

Eat meat

Eat sea food

Eat noodles

Eat junk food

Drink water

Drink coffee/tea

Drink carbonated drinks

Smoke

Exercise

Sleep

**What is the current arrangement for meals?**

(you may select more than 1 option)

*

Prepare myself

Prepare with friends

Buy from shop

Order online/telephone delivery

Parents/relatives cook at home

Prepared by residency/hostel

**Lastly, tell us about your main concerns during this COVID-19 and Lock-down period**

If you are on a mobile phone, please rotate your display to see the full table

*Yes No Not applicable

Uncertainty in the academic semester/year

Coping with online classes

Inadequate internet/communication facilities

Lectures not competent in delivering class online

Not sure how assessments will be done

Not sure if can complete this semester on time

Not sure if I can finish my internship

Not sure if can graduate on time

Not sure if can get a job after graduation

Running into financial problem

Burden to the family

Uncertainty in the academic semester/year

Coping with online classes

Inadequate internet/communication facilities

Lectures not competent in delivering class online

Not sure how assessments will be done

Not sure if can complete this semester on time

Not sure if I can finish my internship

Not sure if can graduate on time

Not sure if can get a job after graduation

Running into financial problem

Burden to the family

Other concerns ...

Your answer

Your comments on this area of research will be highly appropriated

Your answer
